# Communication Barriers Perceived by Nurses and Patients

**DOI:** 10.5539/gjhs.v8n6p65

**Published:** 2015-09-28

**Authors:** Roohangiz Norouzinia, Maryam Aghabarari, Maryam Shiri, Mehrdad Karimi, Elham Samami

**Affiliations:** 1 School of Paramedical, Alborz University of Medical Sciences, Karaj, Iran; 2 Nursing and Midwifery Care Research Center, School of Nursing and Midwifery, Tehran University of Medical Sciences, Tehran, Iran; 3 School of Nursing and Midwifery, Alborz University of Medical Sciences, Karaj, Iran; 4 School of Public Health, Department of Epidemiology and Biostatistics, Tehran University of Medical Sciences, Tehran, Iran

**Keywords:** communication, barrier, nurse, patient

## Abstract

Communication, as a key element in providing high-quality health care services, leads to patient satisfaction and health. The present Cross sectional, descriptive analytic study was conducted on 70 nurses and 50 patients in two hospitals affiliated to Alborz University of Medical Sciences, in 2012. Two separate questionnaires were used for nurses and patients, and the reliability and validity of the questionnaires were assessed. In both groups of nurses and patients, nurse-related factors (mean scores of 2.45 and 2.15, respectively) and common factors between nurses and patients (mean scores of 1.85 and 1.96, respectively) were considered the most and least significant factors, respectively. Also, a significant difference was observed between the mean scores of nurses and patients regarding patient-related (p=0.001), nurse-related (p=0.012), and environmental factors (p=0.019). Despite the attention of nurses and patients to communication, there are some barriers, which can be removed through raising the awareness of nurses and patients along with creating a desirable environment. We recommend that nurses be effectively trained in communication skills and be encouraged by constant monitoring of the obtained skills.

## 1. Introduction

Communication is a multi-dimensional, multi-factorial phenomenon and a dynamic, complex process, closely related to the environment in which an individual’s experiences are shared. Since the time of Florence Nightingale in 19^th^ century until today, specialists and nurses have paid a great deal of attention to communication and interaction in nursing (Fleischer, Berg, Zimmermann, Wüste, & Behrens, 2009). Effective communication is an important aspect of patient care, which improves nurse-patient relationship and has a profound effect on the patient’s perceptions of health care quality and treatment outcomes (Li, Ang, & Hegney, 2012). Effective communication is the key element in providing high-quality nursing care, and leads to patient satisfaction and health (Cossette, Cara, Ricard, & Pepin, 2005). Effective communication skills of health professionals are vital to effective health care provision, and can have positive outcomes including decreased anxiety, guilt, pain, and disease symptoms. Moreover, they can increase patient satisfaction, acceptance, compliance, and cooperation with the medical team, and improve physiological and functional status of the patient; it also has a great impact on the training provided for the patient (Aghabarari, Mohammadi, & Varvani, 2009).

However, most studies have reported poor nurse-patient relationships (McCabe, 2004; Jangland, Gunningberg, & Carlsson, 2009; Gilmartin, & Wright 2008). Therefore, overall, nurse-patient communication has not led to personal satisfaction (Jangland, Gunningberg, & Carlsson, 2009). This is due to the fact that health care quality is strongly affected by nurse-patient relationship, and lack of communication skills (or not using them) has a negative impact on services provided for the patients. The results of previous studies have shown that nurses have been trained to establish an effective communication; however, they do not use these skills to interact with their patients in clinical environments (Heaven, Clegg, & Maguire, 2006). Similarly, the results of other studies show that nurses and nursing professionals in general, have not made a lot of effort for establishing positive interactions with the patients. Many reported problems are related to the decreased sense of altruism among hospital staff including nurses (Bridges et al., 2013).

Communication pitfalls are 5-10% in general population and more than 15% in hospital admissions (Bartlett, Blais, Tamblyn, Clermont, & MacGibbon, 2008). Hospitalized patients in all ages often experience complex communication needs including mobility, sensory, and cognitive needs as well as language barriers during their stay (Downey & Happ, 2013). Hospitalization is potentially stressful and involves unpleasant experiences for patients and their families. All aspects of care and nursing are of high importance in communication with patients, as the patients consider interaction with the nurses as a key to their treatment. Also, through communication, nurses become familiar with the needs of their patients, and therefore, they can deliver high-quality health care services (Cossette et al., 2005; Sheldon, Barrett, & Ellington, 2006; Thorsteinsson, 2002). Patients with communication disability were three times more likely to experience medical or clinical complications compared to other patients (Bartlett et al., 2008).

Iran is a multicultural country with recognized cultural pluralism. In Iranian religious context, nurses are not allowed to gaze or touch patients of the opposite-sex, except in emergency cases. In addition, although Iranian formal language is Farsi, there are many dialects such as Lurish, Kurdish, and Baluchi, which might act as communication barriers between nurses and patients (Anoosheh, Zarkhah, Faghihzadeh, & Vaismoradi, 2009). In Iran, some communication facilitators and barriers have been reported including low educational preparation, governmental policies, and inappropriate environment as barriers, and religious and cultural norms, role modeling, and previous exposure of patients as facilitators (Rejeh, Heravi-Karimooi, & Vaismoradi, 2011).

The first step in eradicating the problems related to nurse-patient communication is two-sided (nurse and patient) awareness of communication barriers. It is of no doubt that building an effective relationship is dependent on the understanding of both sides of the interaction (Park & Song, 2005). In this study, we aimed to determine the barriers to nurse-patient relationship from the perspective of nurses and patients. Through this evaluation, we can improve the quality of nursing services and increase the satisfaction of patients and their families. We also assessed the barriers to using communication skills by the nurses in nurse-patient interactions.

## 2. Method

### 2.1 Study Design

Cross sectional, descriptive analytic study.

### 2.2 Participants

This study was conducted on nurses and patients of two public hospitals affiliated to Alborz University of Medical Sciences, Karaj, Iran. Simple random sampling method was applied.

### 2.3 Questionnaires

Data were collected via two separate questionnaires for nurses and patients. The reliability and validity of the questionnaires were assessed. Content validity was approved by eight professors of Tehran University of Medical Sciences and Tarbiat Modares University, and reliability was assessed using the split-half method. Pearson’s correlation coefficient between the two halves was calculated, and reliability of patient (correlation coefficient of 0.76) and nurse (correlation coefficient of 0.82) questionnaires were approved.

The questionnaires consisted of two sections. The first part included demographic questions and the second part was concerned with the present barriers to nurses’ use of communication skills. The nurse questionnaire contained 44 items and patient questionnaire consisted of 29 items; each item included 5 options: none, little, average, high, and not included. The participants chose one of the options with regard to the importance of each barrier; “not included” was selected if the subjects had not dealt with such a barrier before.

The barriers were divided to four categories: common barriers between patient and nurse, nurse-related barriers, patient-related barriers, and environmental barriers. To determine the importance of each barrier, the qualitative data were converted to quantitative data and the options were scored as follows: none: 1 score, low: 2 scores, average: 3 scores, and high: 4 scores. The difference in the number of items between patient and nurse questionnaires was related to the number of nurse-related factors. Some items were designed with regard to nurses’ working conditions, and there was a possibility that patients were unaware of these conditions; therefore, these cases were removed from the patient questionnaire, and the remainders were included in both questionnaires.

### 2.4 Data Collection

After obtaining the required approval, nurses and patients in the two hospitals were asked to participate in the study. In order to collect the data, the researchers visited the wards on a daily basis during different shifts. After explaining the study objectives to the nurses and taking informed consents, the questionnaires were given to the participants; after completion, the questionnaires were collected by the researchers.

The nurse sample included nurses of medical, surgical, intensive care unit, and emergency wards. They were working in morning, evening, night, evening and night, and circulating shifts. The sample size was calculated according to the number of nurses in the ward.

The inclusion criteria for the nurse group were as follows: 1) bachelor’s degree (minimum education level), 2) minimum of 6-month working experience, and 3) willingness to participate in the study.

The participants in the patient group were selected based on the inclusion criteria of the patient sample: 1) willingness to participate in the study, 2) ability to establish communication, 3) literacy, and 4) older than 15 years of age. The exclusion criteria for the patients were poor speech ability, hearing difficulty, language impairment following a stroke, and intubation.

The subjects were selected from hospitalized patients. After explaining the objectives of the study, an informed consent was obtained and the patient was given a questionnaire, which was collected after 1 hour. The patient sample was randomly selected from medical, surgical, and emergency wards.

### 2.5 Statistical Analysis

For data analysis, descriptive and inferential statistics (Binomial, Mann-Whitney, and Friedman tests) were used and SPSS version 14 was utilized. P-value less than or equal to 0.05 was considered statistically significant.

## 3. Results

### 3.1 Demographic Characteristics

According to the results, the mean age of the nurses was 30.95 yrs, and the mean working experience was 7.02 yrs. The mean age of the patients was 29.30 yrs and the mean of hospitalization days was 2.3 days. Tables 1 and 2 show the demographic characteristics of the subjects.

**Table 1:** Demographic characteristics of nurses

Variables		N (%)
Gender	Female	67 (95.7)
	Male	3 (4.3)
Marital status	Single	21 (30)
	Married	46 (65.7)
	Divorced	3 (4.3)
Education level	Bachelor	65 (92.7)
	MSc	4 (5.7)
	PhD	1 (1.4)
Work shift	Morning	11 (15.7)
	Evening	2 (2.9)
	Night	4 (5.7)
	Evening and night	3 (3.4)
	Circulating	50 (71.4)
ward	Medical	20 (28.6)
	Surgical	25 (35.7)
	Intensive Care Unit CCU, ICU, Dialysis	14 (20)
	Emergency	11 (15.7)
Overtime work	Yes	54 (77.1)
	No	16 (22.9)
Knowledge of communication skills	Yes	56 (80.0)
	No	**14 (20.0)**
Training of communication skills	Trained	**29 (41.4)**
	Untrained	**41 (58.6)**

**Table 2:** Demographic characteristics of patients

Variables		N (%)
Gender	Female	**35**
	Male	**15**
Marital status	Single	**3 (6.0)**
	Married	**47 (94.0)**
Education	Primary	**10 (20.0)**
	Secondary	**14 (18.0)**
	Diploma	**16 (32.0)**
	University	**10 (20.0)**
Hospitalization ward	Medical	**26 (52.0)**
	Surgery	**20 (40.0)**
	Emergency	**4 (8.0)**

### 3.2 The Most and Least Important Barriers

The findings suggest that among four categories of communication barriers in nurse and patient groups, nurse-related factors (mean scores of 2.45 and 2.15, respectively) and common factors between nurses and patients (mean scores of 1.85 and 1.96, respectively) were the most and least important factors, respectively.

### 3.3 Compare the Nurses and Patients’ Viewpoint

Regarding patient-related factors (P=0.001), nurse-related factors (P=0.012) and environmental factors (P=0.019), there was a significant difference between the mean scores of nurses and patients (Table 3).

**Table 3:** Comparison of four groups of barriers in two groups of nurses and patients

Barriers	Nurse group	Patient group	P-value (Mann-Whitney Test)
Factors common between nurses and patients	**1.85±0.54**	**1.96±0.62**	**0.19**
Nurse-related factors	**2.45±0.42**	**2.15±0.61**	0.012*
Patient-related factors	**2.30±0.48**	**1.97±0.53**	0.001**
Environmental factors	**2.41±0.43**	**2.08±0.64**	0.019*

* P≤0.05 was considered statistically significant.

** P≤0.01 was considered statistically significant.

The most frequent communication barriers from the nurses’ viewpoint were as follows: differences in colloquial languages of nurses and patients, nurses’ being overworked, family interference, and presence of emergency patients in the ward. According to the patients, gender differences between nurse and patient, nurse’s reluctance for communication, hectic environment of the ward, and patient’s anxiety, pain, and physical discomfort were the most important barriers to communication (Table 4).

**Table 4:** The most important barriers from the viewpoint of nurses and, based on the type of barrier

Barriers from the viewpoint of nurses	Importance degree	Barriers from the viewpoint of patients	Importance degree
**Factors common between nurses and patients** -Colloquial language differences between nurse and patient -Cultural differences between nurse and patient -Gender differences between nurse and patient	**1** **2** **3**	**Factors common between nurses and patients** -Gender differences between nurse and patient -Cultural differences between nurse and patient -Colloquial language differences between nurse and patient -Age differences between nurse and patient	**1** **2** **2** **3**
**Nurse-related factors** **-Being overworked** **-Shortage of nurses** **-Lack of time**	**1** **2** **3**	**Nurse-related factors** **-Reluctance to communicate with the patient** **-Lack of understanding of the needs and status of the patient** **-Being overworked**	**1** **2** **3**
**Patient-related factors** **- Family interference** **-Patient’s unawareness of the status and duties of the nurse** **-Anxiety, pain, and physical discomfort of the patient**	**1** **2** **3**	**Patient-related factors** **-Anxiety, pain, and physical discomfort of the patient** **-Patient’s lack of attention and focus** **- Patient’s attendees interference**	**1** **2** **3**
**Environmental factors** **-Critically ill patients in the ward** **-Hectic environment of the ward** **-Unsuitable environmental conditions**	**1** **2** **3**	**Environmental factors** **-Hectic environment of the ward** **-Critically ill patients in the ward** **-Unsuitable environmental conditions**	**1** **2** **3**

According to the data obtained from Man Whitney Test, comparison of patients’ and nurses’ mean scores of barriers (to using communication skills by nurses) indicated that of 29 items common between nurse and patient questionnaires, the mean scores of 13 items were significantly different (Table 5).

**Table 5:** Comparison of the mean scores of barriers to communication skills by nurses in interacting with patients from the viewpoint of nurses and patients

Barrier categories	Barriers	M±SD		P-value (Man Whitney)

		Nurses	Patients	
Factors common between nurses and patients	Age differences between nurse and patient	1.30±0.84	1.92±0.89	0.001**
	Gender differences between nurse and patient	1.90±1.0	2.2±1.1	0.045*
	Cultural differences between nurse and patient	2.19±0.80	2.0±0.97	**0.638**
	Religious differences between nurse and patient	1.47±0.89	1.58±1.14	**0.601**
	Colloquial language differences between nurse and patient	2.35±0.68	2.00±0.97	**0.060**
Nurse-related factors	Despondency and apathy of the nurse towards his/her profession	2.6±0.65	2.34±1.01	**0.394**
	Nurse’s lack of knowledge regarding communication skills	2.25±0.72	2.28±0.89	**0.508**
	Nurse’s low self-esteem	2.38±0.85	1.96±1.05	0.024*
	Negative attitude of the nurse towards the patient	2.28±0.90	2.15±1.13	**0.834**
	Nurse’s reluctance to communicate with the patient	2.40±0.84	2.48±0.86	0.451
	Nurse’s insufficient knowledge about the needs and status of the patient	2.27±0.92	2.44±0.93	0.170
	Nurse’s unpleasant experiences of previous encounters with patients	2.24±0.74	1.69±1.01	0.003**
	Relationship between other health care team members and the nurse	2.16±0.77	2.00±0.87	**0.306**
	Shortage of nurses	2.67±0.68	2.30±0.90	0.019*
	Being overworked during the shift	2.70±0.53	2.35±0.91	**0.056**
	Lack of enough time	2.62±0.64	2.04±1.03	0.001**
	Working multiple jobs and fatigue	2.44±0.75	2.06±1.14	**0.105**
	Poor economic status of the nurse	2.36±0.76	1.50±1.24	0.000**
Patient-related factors	Patient’s unawareness of the status and duties of the nurse	2.30±0.75	1.90±0.93	0.025*
	Negative attitude of the patient toward the nurse	2.27±0.79	1.90±0.90	0.023*
	Resistance and reluctance of the patient to communicate	2.28±0.64	1.60±1.11	**0.044**
	Patient’s lack of focus	2.28±0.68	2.02±0.91	**0.154**
	Anxiety, pain, and physical discomfort of the patient	2.30±0.71	2.32±0.87	**0.578**
	family’ interference	2.39±0.69	1.90±1.11	0.026*
	Patient’s companions	2.06±0.76	2.02±1.02	**0.209**
Environmental factors	Unfamiliar environment of the hospital for the patient	2.06±0.76	1.79±1.09	**0.284**
	Busy environment of the ward (noise and traffic)	2.59±0.60	2.23±0.96	**0.070**
	Unsuitable environmental conditions (improper ventilation, heating, cooling, and lighting)	2.48±0.72	1.98±1.10	0.020*
	Critically ill patients in the ward	2.78±0.48	2.17±0.99	0.000**

* P≤0.05 was considered statistically significant.

** P≤0.01 was considered statistically significant

## 4. Discussion

The results of this study showed that in both groups of nurses and patients, the most and the least important barriers were nurse-related factors and common factors between nurses and patients, respectively. These results were consistent with the findings of Aghabarari et al., who performed a study to determine the barriers to applying communication skills by nurses, from the viewpoint of nurses and patients (Aghabarari et al., 2009). Also, in the study of Aghamolaei et al., nurse- and patient-related barriers were more important than environmental barriers (Aghamolaei & Hasani, 2011). In terms of common factors between nurses and patients, colloquial language, and cultural and gender differences were of high importance; however, priorities were not quite similar between nurses and patients. Through establishing an appropriate verbal communication, the nurse could thoroughly understand the patient’s problems; hence, in many studies, the nurse’s unfamiliarity with the patient’s colloquial language has been mentioned as a communication barrier (Anoosheh et al., 2009; Baraz, Shariati, Alijani, & Moein, 2010; del Pino, Soriano, & Higginbottom, 2013; Li et al., 2012). If there is a difference in spoken language, effective communication cannot be established; even non-verbal communication in different cultures may have different interpretations. Patients are also less acceptant of nurses with different languages and cultures (culture has an impact on individuals’ attitudes and behaviors). Based on previous studies, communicative needs and ways of expressing emotions vary in different cultures and religions. Sufficient knowledge of nurses regarding patients’ culture, language, customs, and beliefs can help them communicate with the patients without having any pre-judgments or prejudice. Indeed, culture can act as both a facilitator and a barrier to communication (Okougha & Tilki, 2010).

Alborz province is also called “little Iran”, meaning that all ethnicities and cultures are present in this region; therefore, the variety of cultures should not be neglected. Considering the population concentration of specific ethnic groups in particular regions of this province, it is recommended that the patient’s language and ethnicity be considered for the allocation of medical staff.

In this study, another factor affecting communication, particularly from the viewpoint of patients, was gender differences; the results were consistent with those of previous studies (Anoosheh et al., 2009; Baraz et al., 2010). The impact of gender differences on communication is mostly emphasized by the patients; in fact, nurses are less affected by patients’ gender while performing their professional duties. According to cultural and religious beliefs in Iran, touching and gazing are inconsistent with the principles of the society (Anoosheh et al., 2009). Similar to many Asian cultures, speaking about sexual problems is also considered impolite (Im et al., 2008). Given the aforementioned principles, the number of male nursing students should increase considering the shortage of male nurses in hospital wards.

As to the patients’ viewpoint, another influential factor was age differences. Similarly, Sung and Park considered generation gap as a communication barrier (Park & Song, 2005). In another study, differences in age and social class were included as communication barriers (Anoosheh et al., 2009). However, according to a study by Baraz, age differences had no negative impact on nurse-patient relationship (Baraz et al., 2010). Generally, communicating with different age groups has its own challenges and complexities. Nurses can have good interactions with patients through developing awareness of each age group’s attitudes to health, disease, and body function (Bridges et al., 2013).

Evaluation of the viewpoints of nurses and patients showed that among nurse-related barriers, being overworked, shortage of nurses, and lack of time were the most important barriers for the nurse group. Also, the nurses’ unwillingness to communicate, and lack of understanding of patients’ needs were the most important barriers from the patients’ perspective. Shortage of nurses increases the work load, and therefore, there is not enough time to establish a good therapeutic relationship (Park & Song, 2005); also, nurses’ low income has been mentioned as a barrier to nurse-patient interaction (Aghamolaei & Hasani, 2011; Baraz et al., 2010; Mendes, Trevizan, Nogueira, & Sawada, 1999). Stress, being overworked, and lack of welfare facilities could decrease nurses’ satisfaction and quality of health care provision (Nayeri, Nazari, Salsali, & Ahmadi, 2005). Based on the results of the study by Park and Song, being overworked is a nurse-related communication barrier, which affects the quality and quantity of the relationship between nurses and patients (Park & Song, 2005).

In the present study, comparison of the viewpoints of nurses and patients regarding patient-related barriers showed that interference by family, patients’ unawareness of the status and duties of the nurses, patients’ physical pain, discomfort, and anxiety, lack of attention, and the presence of patients’ companions were the most important factors; these results were considerably consistent with previous studies (Aghabarari et al., 2009).

Definitely, the patient’s disease and dependence after hospitalization result in anxiety, tension, and fear in the patient; moreover, the patient’s family also experiences a difficult time and has different needs and demands. Negligence of the patient and the family of the status and duties of nurses leads to misconceptions about the nurses’ role in the improvement or deterioration of the patient’s health and may even result in the patient’s death. If nurses are not successful in establishing an effective communication with the patients, they can apply communication facilitators; if they still do not succeed, they can explain the problems to the patients so that they can obtain positive treatment results without having a good communication (Ammentorp, Sabroe, Kofoed, & Mainz, 2007).

Nurse is considered the direct care provider and the smallest delay in care provision will be considered as medical negligence. However, it is quite obvious that a medical team consisting of physicians, nurses, clinical departments, and even medical center crew are all responsible for the patient’s health care. Thus, given the direct relationship between nurses and patients, the image created by nurses affects their being accepted as professional staff, and their role will be highlighted in establishing an effective communication (Aghabarari et al., 2009).

In terms of environmental barriers, the presence of critically ill patients in the ward, the hectic environment of the hospital, and unsuitable environmental conditions are considered the main barriers in both groups. The findings of previous studies confirm the aforementioned results. The shortage of nurses and the presence of critically ill patients in the ward cause a lot of stress for the patient and lead to decreased ability and motivation to communicate with other patients; on the other hand, medical environment conditions have great effects on the quantity and quality of communication (Bartlett et al., 2008). Factors disturbing the communication process can be improper temperature, excessive noise, poor ventilation, and lack of respect for the privacy of the two sides of the relationship (Mendes et al., 1999). Thus, providing a safe and comfortable environment leads to psychological and physical comfort of the nurse and patient, and facilitates using communication skills and establishing an effective communication.

One of the limitations of this study was the low number of male nurses. Another limitation was lack of evaluation of cultural forces operating between patients and nurses, regardless of the country of origin or background in the hospitals (active, passive, or power relationships). It is recommended that future studies pay more attention to communication facilitators and divide the participants to male and female groups. It is also suggested that religious and cultural beliefs as well as language barriers be more thoroughly evaluated in patients and nurses.

## 5. Conclusion

The purpose of any system is to provide services with optimal quality and quantity, and health care systems are no exception. One of the best ways to gain the patients’ satisfaction, as major clients of health care systems, is through establishing effective and appropriate communication. Thus, according to the results of this study and previous studies, the following measures will be considerably helpful in establishing an effective nurse-patient communication: allocation of medical staff with regard to the language and culture of the region, motivating nurses to provide high-quality health care services, upgrading medical clinics and facilities, holding periodic workshops of communication skills, holding nursing quality assurance committees, and most importantly, changing attitudes of nursing managers and administrators from offering task-based services toward following a holistic approach.
